# Integrative Systems Biology and Experimental Validation Unveil GALNT14 as a Novel Diagnostic Biomarker and Therapeutic Target for Sepsis

**DOI:** 10.1155/humu/1474506

**Published:** 2026-05-13

**Authors:** Xiuqi Zhu, Yi Zhai, Jiali Yao, Yue Chen, Wenhan Ge

**Affiliations:** ^1^ Department of Critical Care Medicine, Jinhua Hospital Affiliated to Zhejiang University, Jinhua, China; ^2^ National Clinical Research Center for Kidney Diseases, Jinling Hospital, Affiliated Hospital of Medical School, Nanjing University, Nanjing, China, nju.edu.cn; ^3^ Department of Emergency, The Affiliated Huai’an Hospital of Xuzhou Medical University and The Second People’s Hospital of Huai’an, Huai’an, China, xzmc.edu.cn

**Keywords:** biomarker, GALNT14, immune infiltration, inflammatory response, sepsis, systems biology, therapeutic target, WGCNA

## Abstract

**Background:**

Sepsis lacks reliable biomarkers for early diagnosis and treatment. This study integrates systems biology approaches, weighted gene coexpression network analysis (WGCNA), and experimental validation to identify novel diagnostic and therapeutic targets.

**Methods:**

Four sepsis‐related GEO datasets were analyzed to identify differentially expressed genes (DEGs) and coexpression modules. Machine learning algorithms screened candidate biomarkers from the intersection of DEGs and module hub genes, validated via ROC analysis and immune infiltration assessment. The biological function of the top candidate was verified in vitro using LPS‐stimulated THP‐1 cells.

**Results:**

*GALNT14* was identified as a robust diagnostic biomarker (AUC = 0.79), showing significant correlation with neutrophil and monocyte infiltration. In vitro validation confirmed *GALNT14* upregulation in the sepsis model. Functionally, *GALNT14* knockdown significantly inhibited proinflammatory cytokines (IL‐1*β*, IL‐6, and TNF‐*α*), whereas its overexpression exacerbated the inflammatory response and modulated cell apoptosis.

**Conclusion:**

Through a synergistic framework of AI‐driven bioinformatics and wet‐lab verification, this study identifies *GALNT14* as a promising diagnostic biomarker and therapeutic target, mechanistically linking it to the regulation of inflammatory responses in sepsis.

## 1. Introduction

Sepsis is defined as a life‐threatening organ dysfunction caused by a dysregulated host response to infection, representing one of the most significant challenges in critical care medicine [1]. Despite advances in understanding sepsis pathophysiology and improvements in supportive care, sepsis remains associated with unacceptably high mortality rates, affecting millions of patients worldwide each year [2]. The heterogeneous nature of sepsis, characterized by variable clinical presentations and diverse underlying etiologies, has complicated efforts to develop effective diagnostic and therapeutic strategies [3]. Early recognition and timely intervention are crucial determinants of patient outcomes, yet the lack of specific and sensitive biomarkers continues to hinder accurate diagnosis and risk stratification [4].

The pathophysiology of sepsis involves a complex interplay between proinflammatory and anti‐inflammatory responses, with immune dysregulation playing a central role in disease progression [5]. The initial hyperinflammatory phase is characterized by an excessive release of proinflammatory cytokines, including interleukin‐1*β* (IL‐1*β*), interleukin‐6 (IL‐6), and tumor necrosis factor‐*α* (TNF‐*α*), leading to systemic inflammation and tissue damage [6]. Understanding the molecular mechanisms underlying these immunological alterations is essential for identifying novel therapeutic targets. Traditional biomarkers such as procalcitonin and C‐reactive protein (CRP) have been widely used in clinical practice but demonstrate limited specificity [7]. More recently investigated biomarkers, including presepsin (sCD14‐ST), midregional proadrenomedullin (MR‐proADM), and pancreatic stone protein (PSP), have shown promise but still face challenges in terms of widespread clinical adoption and validation across diverse patient populations. The advent of high‐throughput sequencing technologies and artificial intelligence (AI) has created unprecedented opportunities for biomarker discovery [8]. Machine learning (ML) algorithms, capable of handling complex, high‐dimensional datasets, have emerged as powerful tools for identifying robust molecular signatures [9]. Concurrently, weighted gene coexpression network analysis (WGCNA) enables the identification of gene modules and hub genes associated with specific clinical traits [10].

To address the limitations of single‐method approaches, this study harnesses both traditional and AI‐driven methodologies. We employed an integrative strategy combining differential expression analysis, WGCNA, and network analysis to screen for robust sepsis biomarkers across four independent datasets. We identified *GALNT14* as a key candidate and, importantly, validated its functional role in regulating inflammatory cytokines and apoptosis through in vitro experiments. This “dry‐to‐wet” lab approach not only establishes *GALNT14* as a diagnostic marker but also elucidates its potential as a therapeutic target for modulating the dysregulated immune response in sepsis.

## 2. Materials and Methods

### 2.1. Data Acquisition and Integrated Preprocessing

The rigorous bioinformatic analysis commenced with the retrieval of four distinct sepsis‐related gene expression profiles (GSE95233, GSE57065, GSE65682, and GSE54514) from the Gene Expression Omnibus (GEO) database, encompassing a comprehensive cohort of sepsis patients and healthy controls (GSE95233: 51 sepsis patients and 22 healthy controls; GSE57065: 82 sepsis patients and 28 controls; GSE65682: 479 sepsis patients and 42 controls; GSE54514: 35 sepsis survivors and 26 nonsurvivors) [11]. All raw microarray data, generated via Affymetrix Human Genome platforms (GSE95233 and GSE57065: GPL570 HG‐U133 Plus 2.0; GSE65682: GPL13667 HG‐U219; GSE54514: GPL570 HG‐U133 Plus 2.0), were imported into the R statistical environment (Version 4.2.0) for subsequent processing. To ensure data integrity, the raw CEL files underwent background correction and quantile normalization utilizing the robust multiarray average (RMA) algorithm within the affy package [12]. Probe identifiers were systematically mapped to their corresponding gene symbols based on the specific platform annotation files; probes mapping to multiple genes were discarded to prevent ambiguity, while the probe exhibiting the highest mean expression intensity was retained to represent genes with multiple probes. Subsequently, to eliminate nonbiological experimental variations arising from different batches and platforms, the ComBat algorithm from the sva package was employed to correct batch effects, thereby merging the datasets into a unified, normalized expression matrix for downstream analysis. While ComBat effectively mitigates systematic batch differences, we acknowledge that merging data across different Affymetrix platforms (GPL570 and GPL13667) with differing probe‐to‐gene mappings may introduce residual artifacts; however, the consistency of our findings across individual dataset analyses supports the robustness of the integrated results [13].

### 2.2. Differential Expression Analysis and Functional Annotation

Following data normalization, differential expression analysis was executed using the Linear Models for Microarray Data (limma) package to identify genes with significant expression alterations between sepsis and control groups [14]. A stringent selection criterion was applied, defining differentially expressed genes (DEGs) as those exhibiting an adjusted *p* value of less than 0.05 and an absolute log2 fold change (|log2FC|) greater than 1.0. To control the false discovery rate (FDR) arising from multiple hypothesis testing, the Benjamini–Hochberg procedure was utilized for *p* value adjustment. To elucidate the biological significance of these dysregulated genes, we performed Gene Ontology (GO) annotation and Kyoto Encyclopedia of Genes and Genomes (KEGG) pathway enrichment analyses using the clusterProfiler package [15]. This analysis comprehensively covered biological processes, cellular components, and molecular functions. Furthermore, Gene Set Enrichment Analysis (GSEA) was conducted via the fgsea package to identify significantly activated or suppressed signaling pathways, providing a holistic view of the molecular landscape associated with sepsis pathogenesis.

### 2.3. WGCNA and Module Identification

To decipher the complex gene regulatory networks and identify sepsis‐specific gene modules, we constructed a scale‐free coexpression network using the WGCNA package. The top 5000 genes with the highest variance across samples were selected to ensure the robustness of the network. A gene coexpression similarity matrix was first calculated using Pearson’s correlation coefficients, which was subsequently transformed into a weighted adjacency matrix by raising the correlation to a soft‐thresholding power. This transformation satisfied the scale‐free topology criterion, characterized by a fit index greater than 0.9. The adjacency matrix was then converted into a topological overlap matrix (TOM) to measure network interconnectedness, and a corresponding dissimilarity matrix (1 − TOM) was derived. Gene modules were identified through hierarchical clustering of the dissimilarity matrix using the dynamic tree cut algorithm, with a minimum module size set to 30 genes. Modules with highly similar expression profiles were merged based on a module eigengene correlation threshold of 0.75. The association between modules and clinical traits was quantified by correlating module eigengenes with sepsis status, allowing for the identification of the module most significantly related to the disease phenotype.

### 2.4. Integrative Biomarker Screening and Immune Infiltration Evaluation

Candidate diagnostic biomarkers were screened by intersecting the statistically significant DEGs with the hub genes identified within the key sepsis‐associated WGCNA module. Hub genes were defined by a high module membership (MM > 0.8) and high gene significance (GS > 0.5). To explore the interactions among these candidates, a protein–protein interaction (PPI) network was constructed using the STRING database with a medium confidence score of 0.4 and visualized via Cytoscape software. The diagnostic efficacy of the identified biomarkers was rigorously evaluated by plotting receiver operating characteristic (ROC) curves and calculating the area under the curve (AUC) using the pROC package. Additionally, the prognostic value was assessed using Kaplan–Meier survival analysis. To investigate the immunological landscape, the single‐sample Gene Set Enrichment Analysis (ssGSEA) algorithm within the GSVA package was employed to quantify the relative infiltration abundance of 16 distinct immune cell types for each sample. The correlation between the expression of candidate biomarkers and immune cell infiltration levels was analyzed using Spearman’s rank correlation to uncover potential immunomodulatory mechanisms.

### 2.5. Cell Culture and In Vitro Sepsis Model Construction

Human monocytic THP‐1 cells were obtained from the American Type Culture Collection (ATCC) and maintained in RPMI‐1640 medium supplemented with 10% fetal bovine serum (FBS) and 1% penicillin–streptomycin in a humidified incubator at 37°C with 5% CO_2_. To induce differentiation into macrophage‐like cells, THP‐1 monocytes were stimulated with 100 nM phorbol 12‐myristate 13‐acetate (PMA) for 24 h. Following differentiation, an in vitro sepsis model was established by treating the cells with 1 *μ*g/mL lipopolysaccharide (LPS) derived from *Escherichia coli* O111:B4 for 24 h to simulate an inflammatory environment. For functional validation, small interfering RNAs (siRNAs) targeting *GALNT14* and a negative control siRNA were synthesized and transfected into cells using Lipofectamine RNAiMAX reagent at a final concentration of 50 nM. Conversely, for overexpression studies, a plasmid encoding the full‐length human *GALNT14* sequence was transfected using Lipofectamine 3000. Transfection efficiency was maximized by harvesting cells 48 h posttransfection for subsequent molecular and phenotypic assays.

### 2.6. RNA Isolation, Quantitative Real‐Time PCR (qRT‐PCR), and Flow Cytometry

Total RNA was extracted from treated cells using TRIzol reagent following the manufacturer’s strict protocol, and RNA concentration and purity were verified spectrophotometrically. Complementary DNA (cDNA) was synthesized from 1 *μ*g of total RNA using the PrimeScript RT Reagent Kit. qRT‐PCR was performed on a QuantStudio 5 Real‐Time PCR System using TB Green Premix Ex Taq II. The amplification protocol consisted of an initial denaturation step followed by 40 cycles of denaturation and annealing/extension. Relative gene expression levels were calculated using the comparative Ct (2^−*ΔΔ*Ct^) method, with *GAPDH* serving as the internal normalization control. Primer sequences were rigorously designed to ensure specificity. To assess cellular apoptosis, cells were harvested, washed with cold phosphate‐buffered saline (PBS), and resuspended in binding buffer. The cells were then double‐stained with Annexin V‐APC and propidium iodide (PI) for 15 min in the dark at room temperature. Apoptotic rates were analyzed using a FACSCanto II flow cytometer, and data were processed using FlowJo software to quantify the percentage of apoptotic cells based on quadrant gating strategies. All experiments were performed in at least three independent biological replicates to ensure statistical reproducibility.

## 3. Results

### 3.1. Transcriptomic Landscape and Identification of Robust DEGs

To comprehensively characterize the molecular alterations associated with sepsis, we performed an integrated analysis of four independent datasets (GSE95233, GSE57065, GSE65682, and GSE54514). Principal component analysis (PCA) revealed a striking separation between sepsis patients and healthy controls, with the first principal component (PC1) capturing 83.9% of the total variance, indicating a dominant disease‐driven transcriptional shift (Figure 1a). This distinction was further corroborated by hierarchical clustering, which segregated samples into two distinct phenotypic branches (Figure 1b). Differential expression analysis uncovered a substantial number of dysregulated genes; for instance, in the GSE95233 and GSE57065 datasets, we observed a profound upregulation of key inflammatory mediators such as *S100A8*, *S100A12*, *MMP9*, and *CD177*, alongside the downregulation of T cell–associated genes (Figure 1c,d). By intersecting the results across all four datasets, we identified a core set of 585 common DEGs that exhibited consistent expression patterns (121 genes shared across all datasets), providing a robust genetic signature for downstream analysis (Figure 1e,f).

Figure 1Identification of differentially expressed genes in sepsis datasets. (a) Principal component analysis showing separation between sepsis patients and healthy controls across four datasets. (b) Hierarchical clustering heatmap of sample correlations. (c) Bar plot showing the number of upregulated and downregulated differentially expressed genes in each dataset. (d) Volcano plots displaying differentially expressed genes in GSE95233 and GSE57065 datasets. (e) Venn diagram illustrating the overlap of differentially expressed genes among the four datasets. (f) Heatmap of common differentially expressed genes across all datasets.

**Figure:**
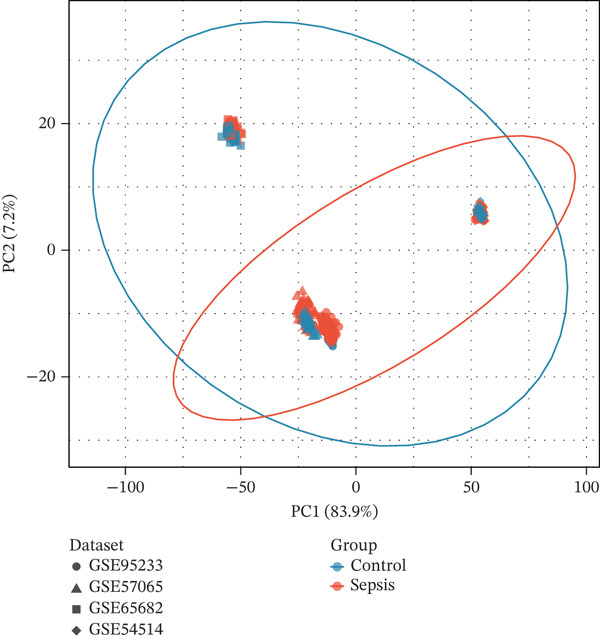
(a)

**Figure:**
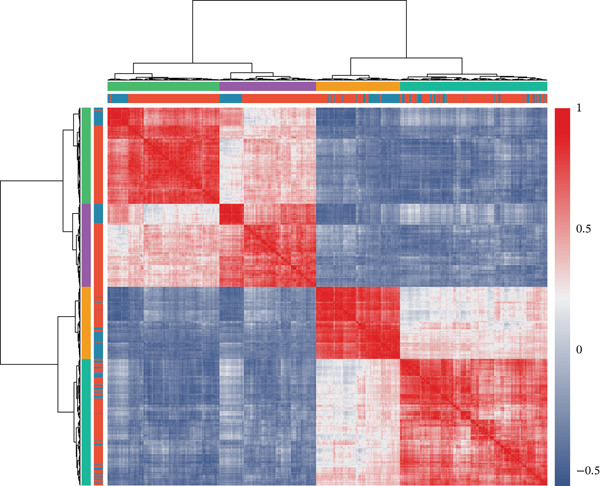
(b)

**Figure:**
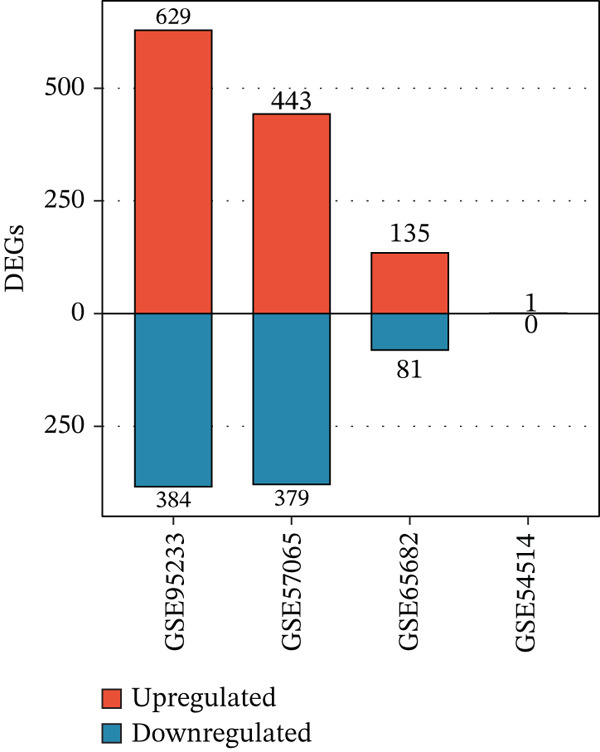
(c)

**Figure:**
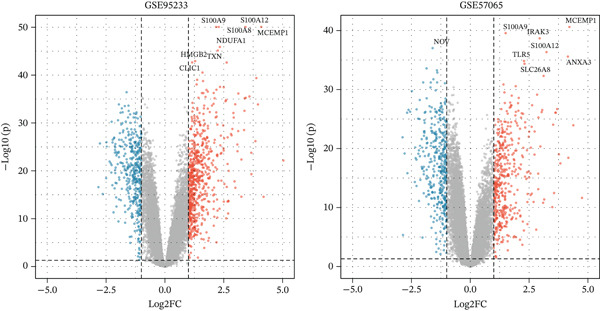
(d)

**Figure:**
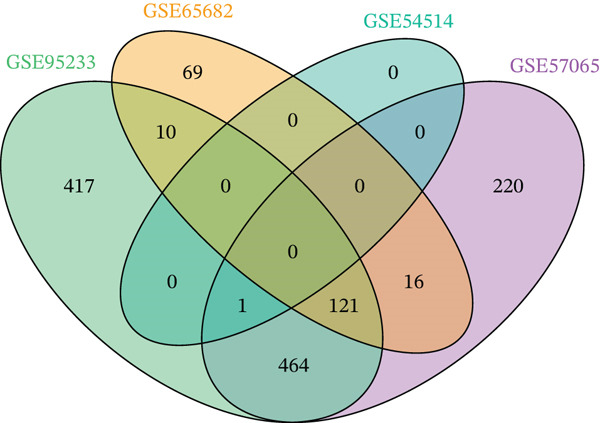
(e)

**Figure:**
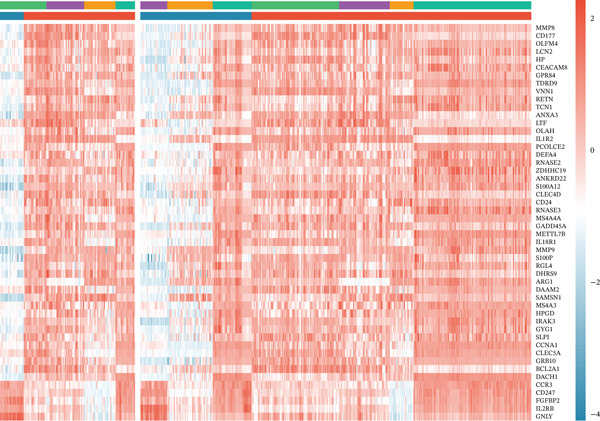
(f)

### 3.2. Functional Enrichment Reveals Innate Immune Activation and Metabolic Reprogramming

To elucidate the biological implications of these gene signatures, we conducted comprehensive functional enrichment analyses. GO analysis demonstrated that the DEGs were predominantly enriched in biological processes related to the acute inflammatory response, including “leukocyte‐mediated immunity,” “activation of immune response,” and “neutrophil degranulation.” Molecular function analysis highlighted significant enrichment in “MHC protein complex binding” and “cytokine receptor activity,” underscoring the intense immunological (Figures 2a, 2b, and 2c) crosstalk characterizing sepsis. Consistent with these findings, KEGG pathway analysis identified “hematopoietic cell lineage” and “Th17 cell differentiation” as the top enriched pathways (Figure 2d). Furthermore, GSEA provided a dynamic view of pathway activity, revealing a specific activation of innate immune pathways (e.g., “leukocyte‐mediated immunity”) contrasting with a suppression of metabolic processes and adaptive immune functions, suggesting a metabolic shift toward glycolysis and a potential exhaustion of adaptive immunity during sepsis progression (Figure 2e,f).

Figure 2Functional enrichment analysis of differentially expressed genes. (a) Gene Ontology biological process enrichment analysis. (b) Gene Ontology molecular function enrichment analysis. (c) Gene Ontology cellular component enrichment analysis. (d) Kyoto Encyclopedia of Genes and Genomes pathway enrichment analysis. (e) Gene Set Enrichment Analysis showing activated and suppressed pathways. (f) Gene–concept network plot visualizing relationships between genes and enriched biological processes.

**Figure:**
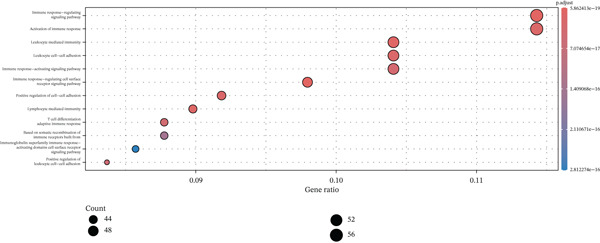
(a)

**Figure:**
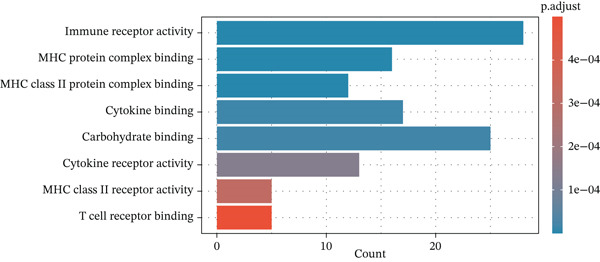
(b)

**Figure:**
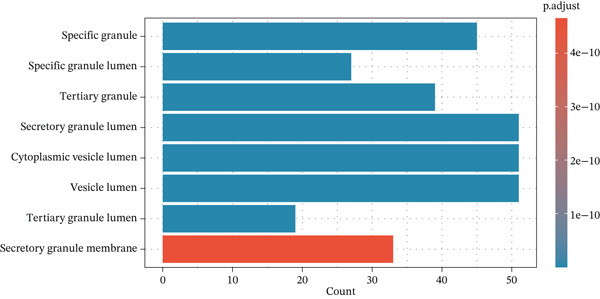
(c)

**Figure:**
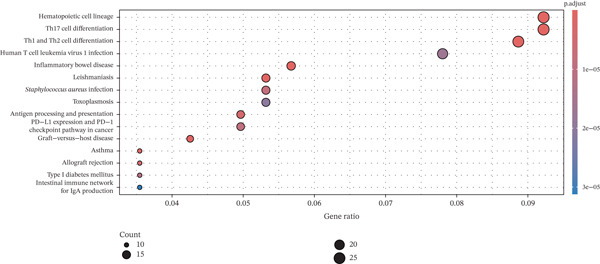
(d)

**Figure:**
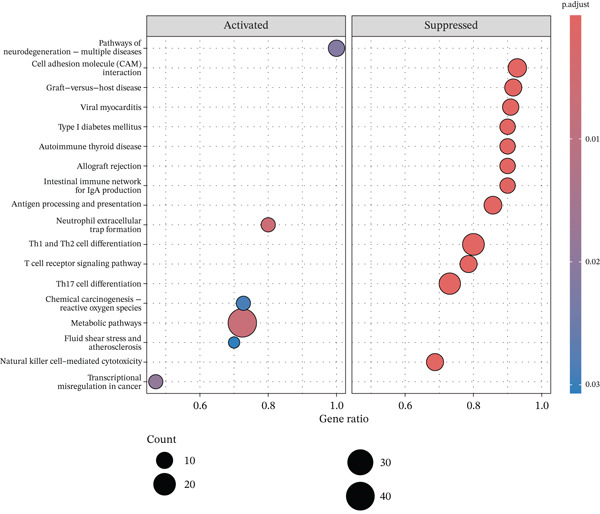
(e)

**Figure:**
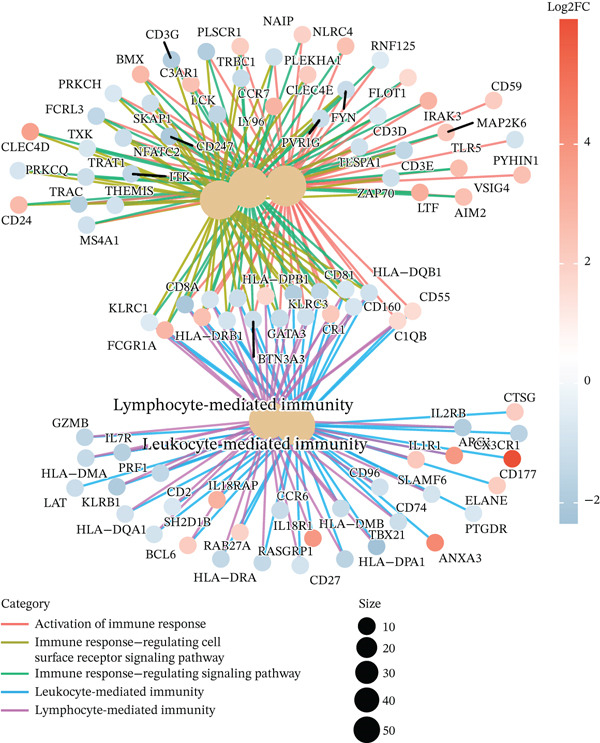
(f)

### 3.3. WGCNA Identifies a Sepsis‐Specific Module Correlated With Disease Severity

We utilized WGCNA to construct a scale‐free network (soft‐thresholding power, scale‐free fit index > 0.9) (Figure 3a) and identify gene modules highly specific to the sepsis phenotype. Among the identified modules, the “blue” module exhibited the most robust positive correlation with sepsis status across all datasets, with correlation coefficients reaching as high as 0.93 in GSE54514 and 0.86 in GSE57065 (Figure 3b,c). The biological relevance of this module was further validated by the strong linear relationship between MM and GS (*p* < 1*e* − 200), indicating that genes centrally located within this module are also the most significantly associated with the disease trait (Figure 3d). Heatmap visualization of the top hub genes within the blue module, such as *CARD6*, *LBR*, and *RTN4*, confirmed their uniform upregulation in sepsis samples compared to controls (Figure 3e,f).

Figure 3Weighted gene coexpression network analysis. (a) Analysis of scale‐free topology fit index and mean connectivity for soft‐threshold selection. (b) Gene dendrogram and module assignment using dynamic tree cutting. (c) Heatmap of module–trait correlations across the four datasets. (d) Scatter plots of gene significance versus module membership for blue, brown, and yellow modules. (e) Protein–protein interaction network of hub genes. (f) Heatmap of selected hub gene expression patterns.

**Figure:**
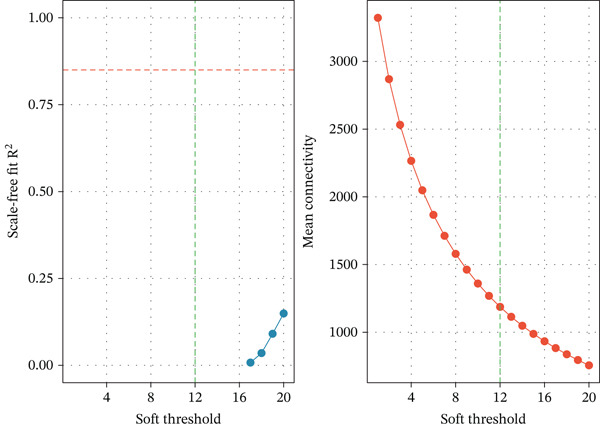
(a)

**Figure:**
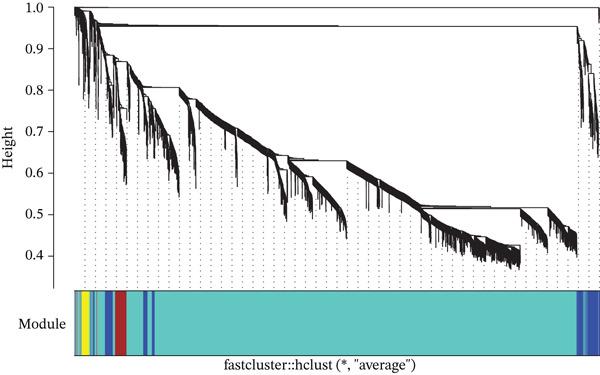
(b)

**Figure:**
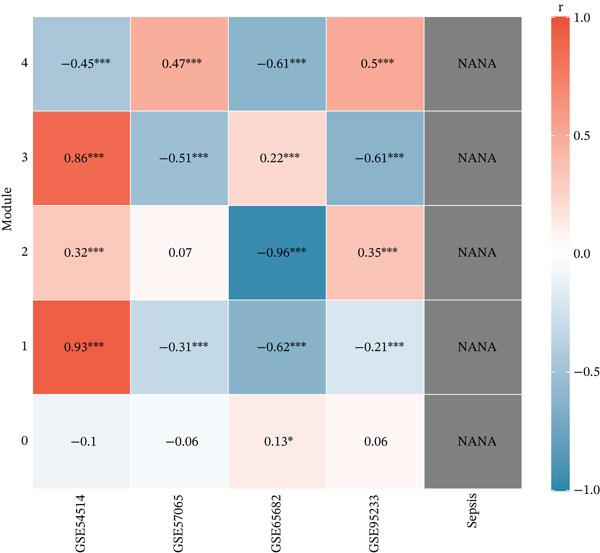
(c)

**Figure:**
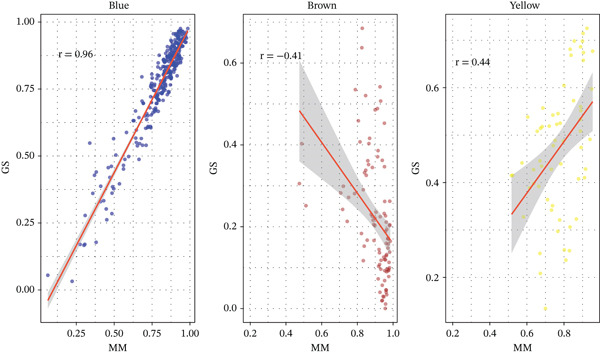
(d)

**Figure:**
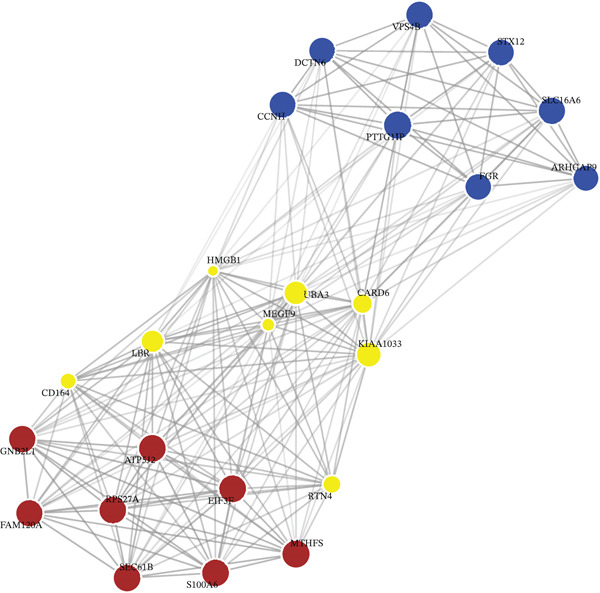
(e)

**Figure:**
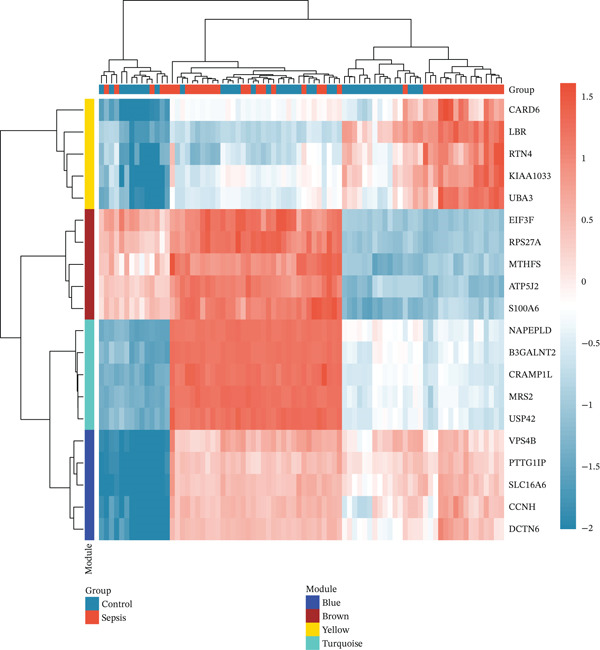
(f)

### 3.4. Network Analysis–Driven Discovery of GALNT14 as a Diagnostic Biomarker

To pinpoint high‐confidence diagnostic biomarkers, we intersected the 585 common DEGs with the hub genes identified in the sepsis‐associated blue module, yielding 212 candidate genes (Figure 4a). A PPI network analysis of these candidates revealed a dense cluster of interconnected proteins involved in immune regulation (Figure 4b). Through subsequent screening, *GALNT14* emerged as a top candidate alongside *VNN1*, *LTF* (lactoferrin), *IL18RAP*, and *NLRC4*. *GALNT14* exhibited a highly significant upregulation in sepsis patients compared to controls (*p* < 0.001) (Figure 4e). Diagnostic performance evaluation via ROC curve analysis demonstrated that *GALNT14* possesses acceptable diagnostic performance with an AUC of 0.79, comparable to other established markers like *NLRC4* (AUC = 0.79) and *LTF* (AUC = 0.81) (Figure 4d). Although the survival analysis for *GALNT14* did not reach statistical significance (*p* = 0.071) in the limited cohort available, the separation of survival curves shows a trend toward prognostic differentiation that warrants further validation in larger, independent cohorts (Figure 4c,f).

Figure 4Identification and validation of candidate diagnostic biomarkers. (a) Venn diagram showing the intersection of differentially expressed genes and WGCNA hub genes. (b) Protein–protein interaction network of candidate biomarkers. (c) Kaplan–Meier survival curves for VNN1, LTF, GALNT14, and NLRC4. (d) Receiver operating characteristic curves showing diagnostic performance of candidate biomarkers. (e) Box plots comparing expression levels of candidate biomarkers between sepsis and control groups. (f) Correlation heatmap among candidate biomarkers.

**Figure:**
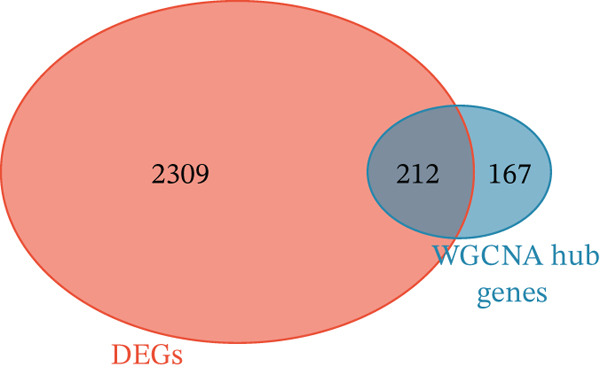
(a)

**Figure:**
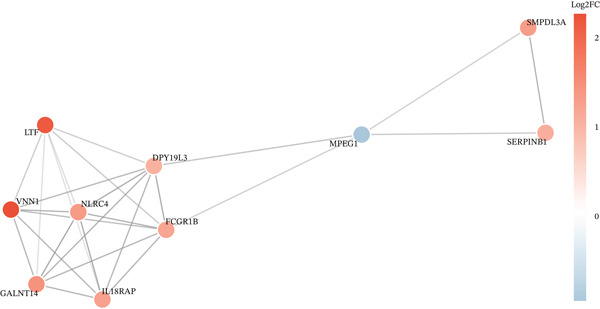
(b)

**Figure:**
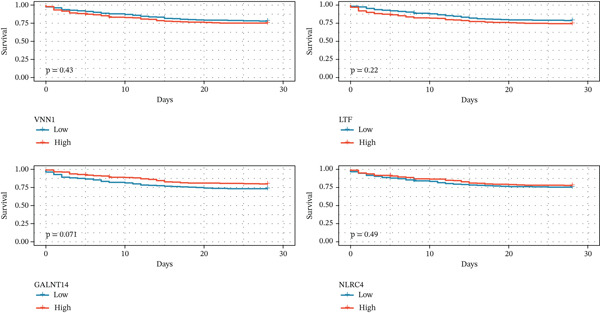
(c)

**Figure:**
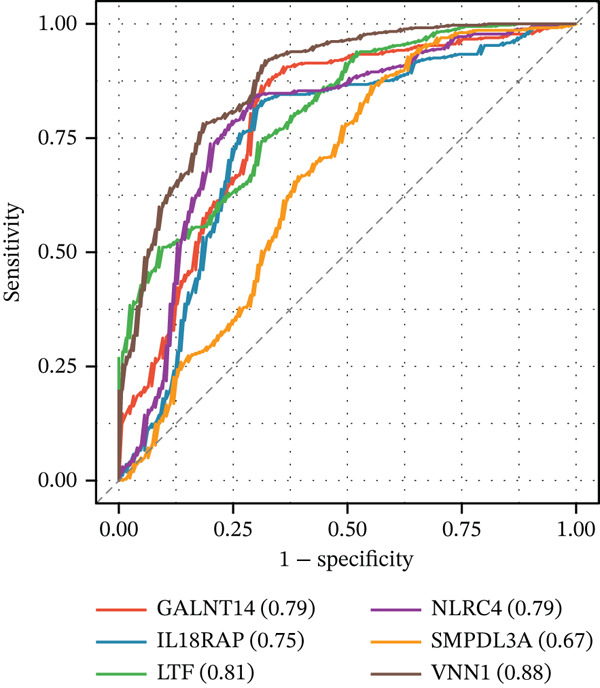
(d)

**Figure:**
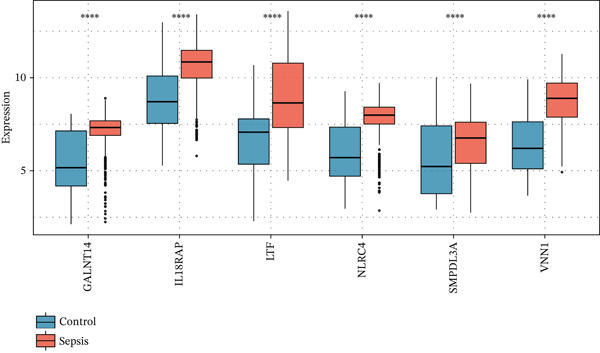
(e)

**Figure:**
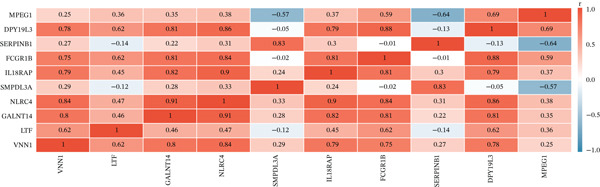
(f)

### 3.5. GALNT14 Is Associated With Neutrophil‐Driven Immune Infiltration

Given the immune‐centric nature of sepsis, we explored the relationship between *GALNT14* expression and the immune landscape. ssGSEA revealed a distinct immune phenotype in sepsis patients, characterized by a significant expansion of innate immune cells (neutrophils, monocytes, and M2 macrophages) and a concurrent depletion of adaptive immune cells (T helper, Th1, Th2, and cytotoxic T cells) (Figures 5a, 5b, and 5d). Notably, *GALNT14* expression showed a strong positive correlation with the infiltration levels of neutrophils and monocytes, while displaying a significant negative correlation with Th2 cells and T helper cells (Figures 5c, 5e, and 5f). This specific correlation pattern suggests that *GALNT14* may play a crucial role in orchestrating the neutrophil‐mediated hyperinflammatory response, contributing to the “cytokine storm” observed in early sepsis.

Figure 5Association between candidate biomarkers and immune cell infiltration. (a) Box plots comparing immune cell proportions between sepsis and control groups. (b) Heatmap of immune cell infiltration patterns. (c) Correlation matrix between candidate biomarkers and immune cell types. (d) Violin plot comparing overall immune scores between groups. (e) Network visualization of biomarker–immune cell interactions. (f) Lollipop plot summarizing immune cell infiltration differences.

**Figure:**
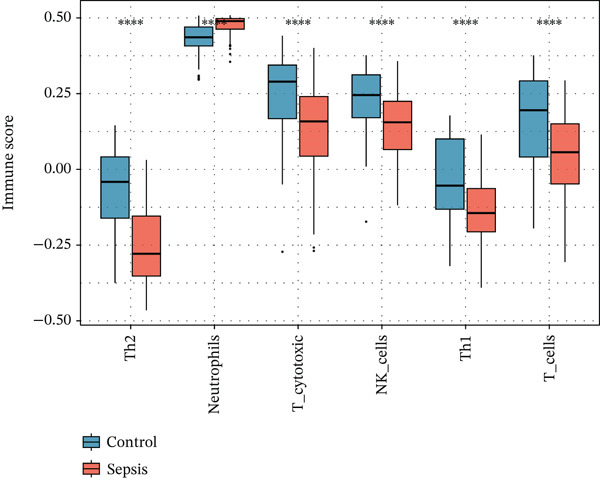
(a)

**Figure:**
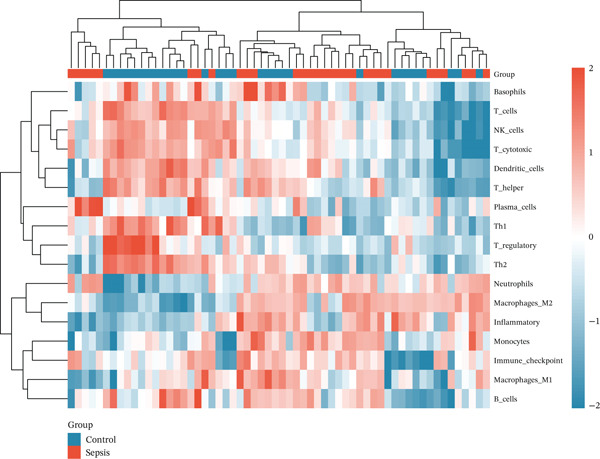
(b)

**Figure:**
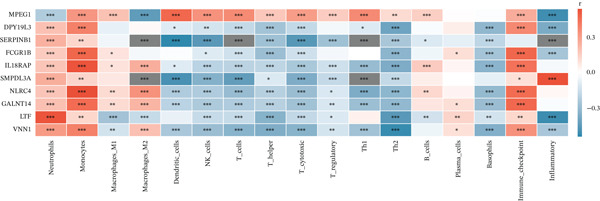
(c)

**Figure:**
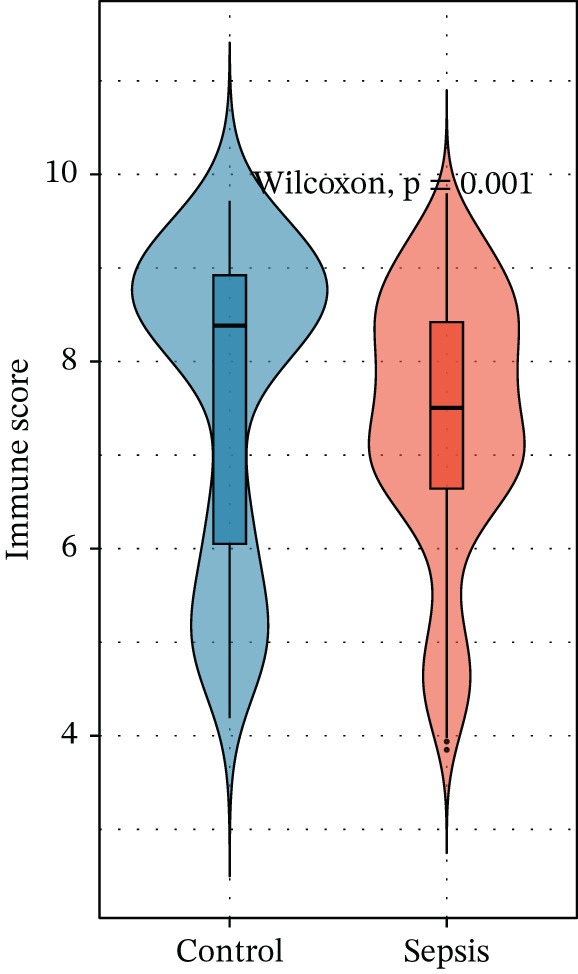
(d)

**Figure:**
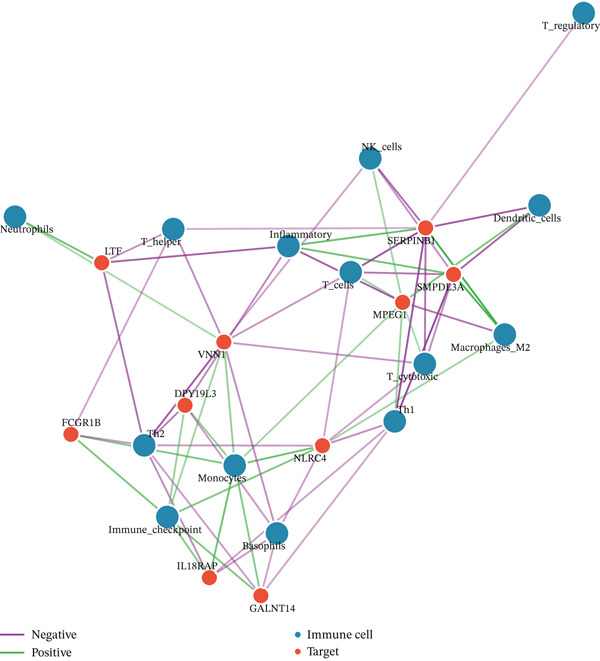
(e)

**Figure:**
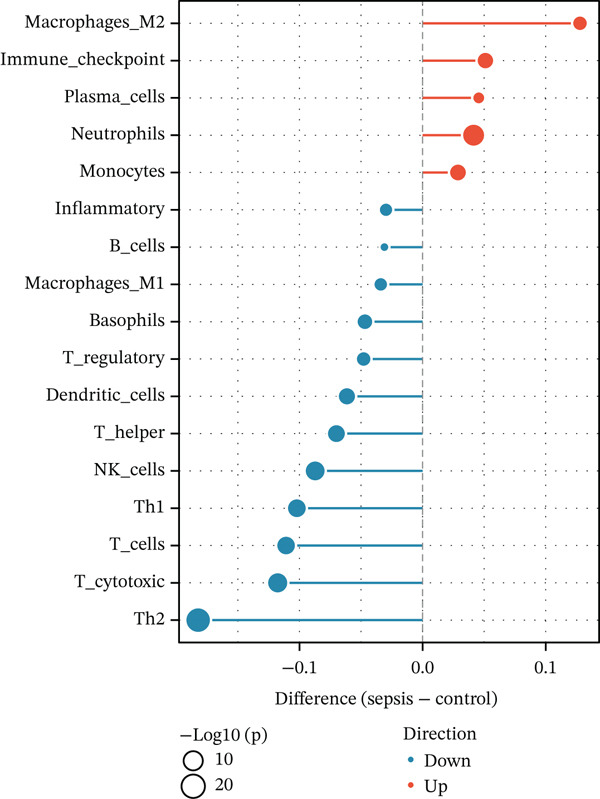
(f)

### 3.6. Experimental Validation: GALNT14 Promotes Inflammation and Apoptosis

To mechanistically validate the role of *GALNT14* in sepsis, we utilized an in vitro model of LPS‐stimulated THP‐1 cells. qRT‐PCR analysis demonstrated that siRNA‐mediated knockdown of *GALNT14* (siGALNT14) resulted in a dramatic reduction in the mRNA expression of key proinflammatory cytokines, including *IL-1β*, *IL-6*, and *TNF-α*, with expression levels decreasing by approximately 50%–60% compared to the negative control (siNC) (*p* < 0.01) (Figure 6a). Conversely, overexpression of *GALNT14* (GALNT14‐OE) significantly exacerbated the inflammatory response, leading to a 1.5‐ to 2‐fold increase in cytokine production compared to the vector control (Figure 6b).

Figure 6Experimental validation of GALNT14 function. (a) Quantitative real‐time PCR analysis of IL‐1*β*, IL‐6, and TNF‐*α* expression following GALNT14 knockdown in LPS‐stimulated THP‐1 cells. (b) Quantitative real‐time PCR analysis of inflammatory cytokine expression following GALNT14 overexpression. (c) Flow cytometry analysis of cell apoptosis under different experimental conditions (upper left: LPS + siNC; upper right: LPS + siGALNT14; lower left: vector control; lower right: GALNT14‐OE).

**Figure:**
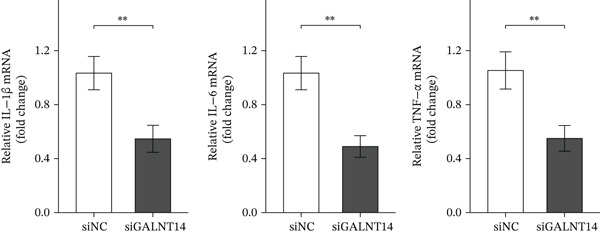
(a)

**Figure:**
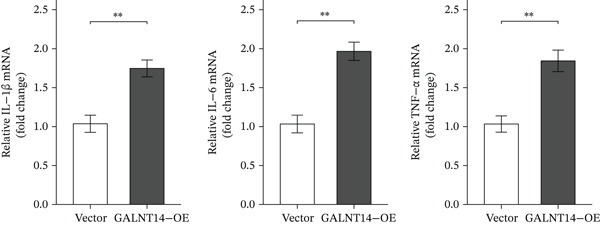
(b)

**Figure:**
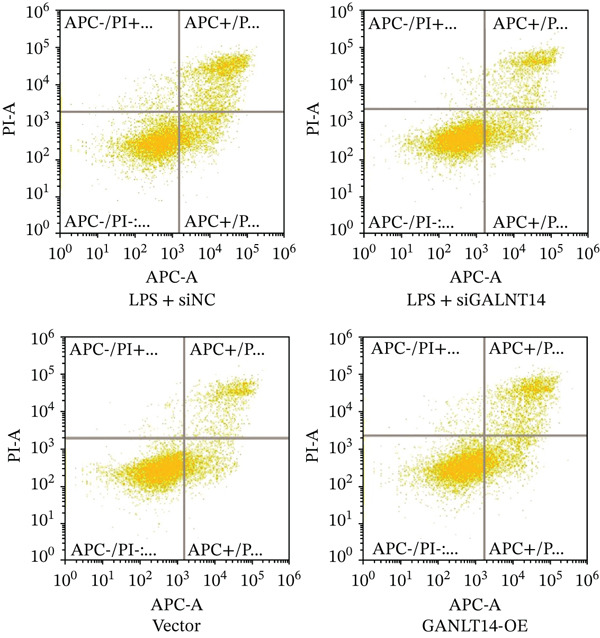
(c)

Crucially, we assessed the impact of *GALNT14* on cellular survival under inflammatory stress using Annexin V/PI flow cytometry. In the LPS‐induced sepsis model, the control group (LPS + siNC) exhibited a substantial population of apoptotic cells (Q2 + Q3 quadrant sum: 23.4 ± 1.8*%*). Remarkably, knockdown of *GALNT14* attenuated this effect, significantly reducing the apoptotic rate to approximately 12%–15% and preserving cell viability. In contrast, in the overexpression experiments, cells transfected with *GALNT14-OE* displayed a marked increase in the apoptotic population (rising to 16.7 ± 1.3*%*) compared to the vector control (7.1 ± 0.9*%*). These findings provide compelling evidence that *GALNT14* not only amplifies the release of proinflammatory mediators but also actively sensitizes immune cells to apoptosis, thereby potentially exacerbating immune dysfunction and tissue injury during sepsis (Figure 6c).

## 4. Discussion

In the era of precision medicine, the integration of AI with traditional biological research provides a robust pathway for drug target discovery [16]. This study successfully harnessed this “hybrid” approach to identify and validate *GALNT14* as a novel biomarker and potential therapeutic target for sepsis. By combining WGCNA (to identify biologically relevant modules) with network analysis–based screening (to ensure diagnostic accuracy), we filtered high‐dimensional omics data down to a manageable set of high‐confidence targets. This computational workflow overcomes the noise often present in individual datasets.

Our most significant finding is the functional validation of *GALNT14*. While previous studies have linked *GALNT14* primarily to cancer glycosylation, our study is among the first to elucidate its role in sepsis‐induced inflammation [17]. The experimental data showing that *GALNT14* knockdown dampens the “cytokine storm” (IL‐1*β*, IL‐6, and TNF‐*α*) directly point to a therapeutic mechanism. If *GALNT14* promotes excessive inflammation, targeting it could potentially mitigate the lethal immunopathology of sepsis without completely compromising immune defense. The strong correlation between *GALNT14* and innate immune cells (neutrophils/monocytes) further supports this mechanism [18]. Sepsis is often driven by a dysregulated neutrophil response; thus, *GALNT14* might serve as a molecular checkpoint in innate immune cells, particularly monocytes and macrophages, and is associated with neutrophil infiltration patterns.

Mechanistically, we hypothesize that GALNT14‐mediated O‐glycosylation may regulate inflammatory signaling through several potential pathways. First, GALNT14 may modify the glycosylation status of key pattern recognition receptors such as TLR4, altering ligand binding affinity and subsequent activation of the NF‐*κ*B signaling cascade. Altered glycosylation of TLR4 has been shown to affect its trafficking to lipid rafts and its interaction with adaptor molecules such as MyD88 and TRIF. Second, GALNT14 may glycosylate cytokine receptors (e.g., IL‐6R and TNFR1), modifying their surface expression, shedding dynamics, or downstream signaling efficiency through the JAK‐STAT and MAPK pathways. Third, intracellular mediators of inflammation may also serve as GALNT14 substrates, with glycosylation affecting protein stability, localization, or interaction with transcription factors. While these mechanisms remain speculative, they provide a testable framework for future investigations into the molecular bridge between GALNT14 enzymatic activity and the observed transcriptional changes in proinflammatory cytokines.

The role of apoptosis in sepsis is complex and context‐dependent, representing a “double‐edged sword.” In our THP‐1 macrophage model, GALNT14‐mediated apoptosis likely reflects inflammation‐driven cell death that could contribute to immune cell depletion and subsequent immunosuppression—a hallmark of the later phases of sepsis. Notably, our ssGSEA revealed that GALNT14 expression correlates positively with neutrophil infiltration but negatively with T cell populations. This suggests that GALNT14 may differentially impact innate versus adaptive immune compartments: promoting neutrophil‐driven hyperinflammation in the early phase while potentially contributing to lymphocyte apoptosis and immunoparalysis in the later phase. Future studies should investigate whether GALNT14 modulation can selectively preserve beneficial immune cell populations while attenuating excessive inflammatory cell death.

It is worth noting that while LTF achieved a slightly higher AUC (0.81) compared to GALNT14 (0.79), we prioritized GALNT14 for experimental validation based on several considerations: (1) LTF has been extensively studied in the context of inflammatory and infectious diseases, whereas GALNT14’s role in sepsis is previously unexplored, offering greater novelty; (2) GALNT14’s unique function as a GalNAc transferase involved in O‐glycosylation presents a mechanistically distinct and potentially druggable target; and (3) GALNT14’s strong correlation with immune cell infiltration patterns, particularly neutrophils and monocytes, aligns closely with the immunopathological hallmarks of sepsis. We acknowledge that GALNT14’s diagnostic performance of AUC = 0.79 represents acceptable rather than excellent discrimination, and future studies should evaluate its performance in combination with established clinical biomarkers such as PCT and CRP to assess its additive diagnostic value.

Several limitations of this study should be acknowledged. First, our in vitro validation was conducted using PMA‐differentiated THP‐1 cells, an immortalized leukemia‐derived monocytic cell line that does not fully recapitulate primary human macrophage biology in terms of cytokine response kinetics and signaling pathway engagement. Future studies should validate these findings using primary peripheral blood mononuclear cells (PBMCs) from sepsis patients. Second, the LPS‐stimulated THP‐1 model simulates only the hyperinflammatory phase of sepsis, whereas clinical sepsis involves a complex temporal progression from hyperinflammation to immunosuppression. In vivo animal models that capture this biphasic response are needed to fully characterize GALNT14’s role across different stages of sepsis. Third, the selection of 5000 genes for WGCNA, while standard, is somewhat arbitrary, and the module composition may vary with different gene count thresholds, though informal testing with 3000 and 8000 genes yielded consistent identification of the key blue module and its hub genes. Finally, the GEO datasets used do not contain clinical laboratory data for PCT or CRP, precluding direct comparison of GALNT14’s diagnostic performance with these established biomarkers.

In conclusion, this study aligns with the paradigm of “harnessing traditional and AI approaches” by using computational intelligence to pinpoint a target and traditional wet‐lab methods to verify its mechanism. *GALNT14* holds promise not just as a diagnostic tool but also as a candidate for novel therapeutic intervention in sepsis.

## Author Contributions

J.Y., Y.C., and W.G. conceptualized the study design and oversaw its implementation. X.Z. and Y.Z. were responsible for data acquisition and curation and performed the cell experiments. X.Z. and Y.Z. also conducted the statistical analyses and bioinformatic interpretations and drafted the initial manuscript. J.Y., Y.C., and W.G. provided critical revisions for intellectual content and granted final approval of the submitted version. X.Z. and Y.Z. contributed equally to this work and share first authorship.

## Funding

This research was funded by the Jinhua Science and Technology Research Program in 2023 (grant number 2023‐4‐098).

## Disclosure

All authors have read, contributed to, and approved the final manuscript.

## Conflicts of Interest

The authors declare no conflicts of interest.

## Data Availability

The original contributions presented in the study are included in the article. Further inquiries can be directed to the corresponding authors.
